# Supplementary material for: TastepepAI: An artificial intelligence platform for taste peptide de novo design
Source: PLoS Comput Biol. 2025 Oct 16;21(10):e1013602. doi: 10.1371/journal.pcbi.1013602 (PMC12543283; doi:10.1371/journal.pcbi.1013602)
Supplement: S1 Text — Fig A. Sequence similarity networks of taste peptides based on global and local alignment algorithms. (A) Global sequence similarity network constructed using the Needleman-Wunsch algorithm with spring layout algorithm for network visualization optimization. (B) Local sequence similarity network constructed using the Smith-Waterman algorithm with Kamada-Kawai layout algorithm to emphasize local sequence similarities. In both networks, nodes represent individual peptides with size proportional to sequence length, and node colors indicate different taste properties. Edge thickness corresponds to the degree of sequence similarity between peptides. Fig B. Length distribution and amino acid composition analysis of toxic and non-toxic peptides. (A) Length distribution of the complete dataset comprising 6861 toxic peptides (red) and 9183 non-toxic peptides (blue). (B) Amino acid frequency distribution in the complete dataset. (C) Length distribution of the filtered dataset (≤25 AA) containing 2821 toxic peptides and 2821 length-matched non-toxic peptides. (D) Amino acid frequency distribution in the filtered dataset (≤25 AA). Fig C. Length-specific amino acid frequency analysis of toxic peptides. Frequency distribution analysis of amino acid residues across different sequence length groups in toxic peptides. Each subplot shows how a specific amino acid’s frequency (%) varies among peptide sequence sets of different lengths (4–50 AA). The vertical dashed line at 25 AA indicates our sequence length threshold for model development. The mean frequency value is indicated for each residue. Fig D. Comparative analysis of amino acid frequencies between shorter (≤25 AA) and longer (26–50 AA) toxic peptides. Frequency distribution (%) of 20 amino acid residues in shorter (≤25 AA, pink) and longer (26–50 AA, blue) toxic peptide sequences. Error bars represent standard errors. Statistical significance levels are indicated (*p < 0.05, **p < 0.01, ***p < 0.001). Eighteen amino acids sho [file pcbi.1013602.s001.docx]

Supplementary Information

**An artificial intelligence platform for complex taste peptide design and safety assessment**

Jianda Yue *^a,c,d^*^,#^, Tingting Li *^a,c,d^*^,#^, Jian Ouyang *^b,e^*, Jiawei Xu *^a,c,d^*, Hua Tan *^a,c,d^*, Zihui Chen *^a,c,d^*, Changsheng Han *^a,c,d^*, Huanyu Li *^a,c,d^*, Songping Liang *^a,c,d^*, Zhonghua Liu (刘中华) *^a,c,d,^*^*^, Zhonghua Liu (刘仲华) *^a,b,c,d^*^,e*^, Ying Wang *^a,c,d^*^,*^

*^a^The National and Local Joint Engineering Laboratory of Animal Peptide Drug Development, College of Life Sciences, Hunan Normal University, Changsha 410081, Hunan, China*

*^b^Key Laboratory of Tea Science of Ministry of Education, Hunan Agricultural University, Changsha 410128, China*

*^c^Peptide and small molecule drug R&D plateform, Furong Laboratory, Hunan Normal University, Changsha 410081, Hunan, China*

*^d^Institute of Interdisciplinary Studies, Hunan Normal University, Changsha 410081, Hunan, China*

*^e^National Research Center of Engineering Technology for Utilization of Functional Ingredients from Botanicals, Hunan Agricultural University, Changsha 410128, China*

^#^The authors give equal contributions.

*Corresponding authors


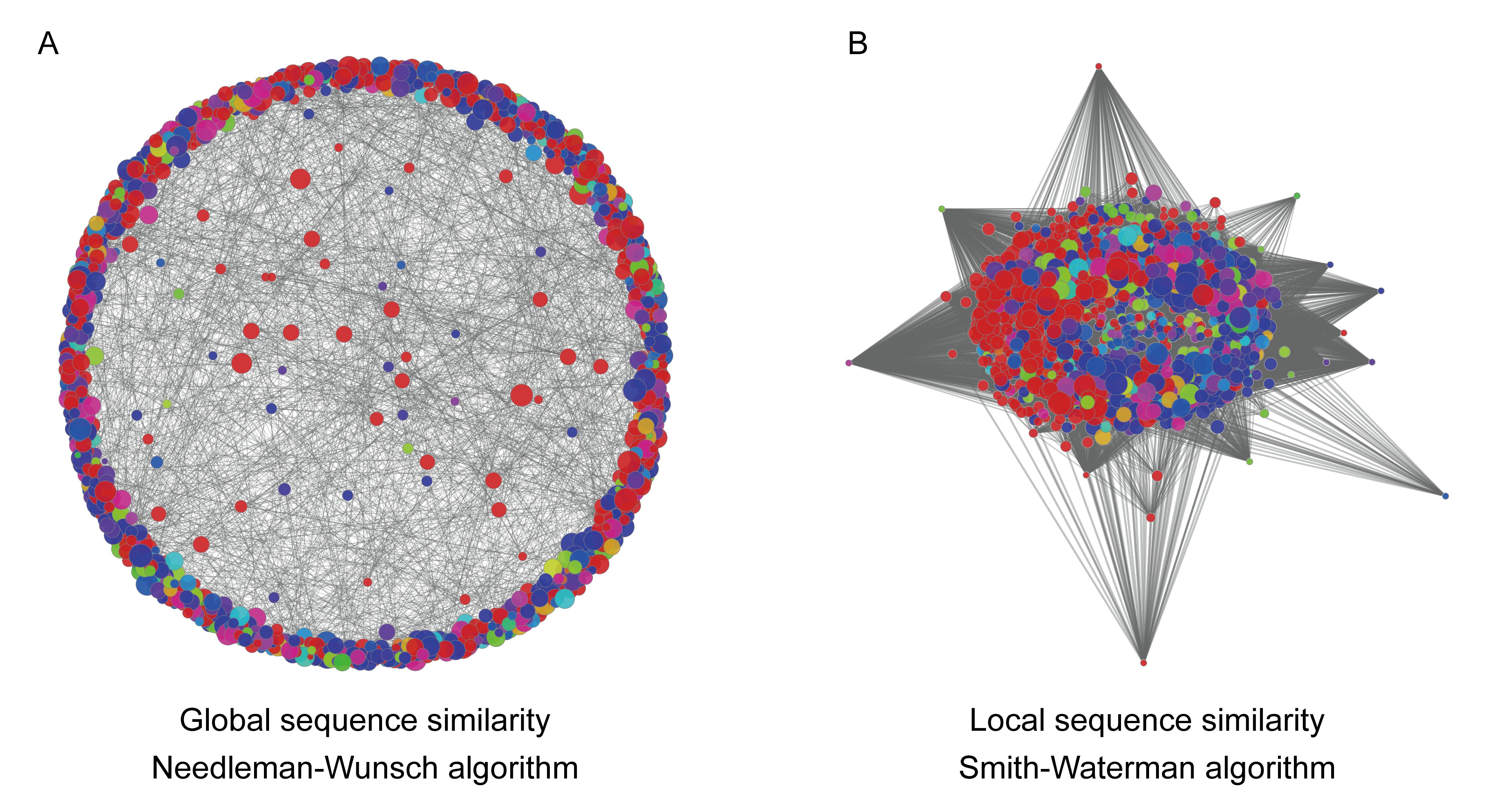


**Fig A. Sequence similarity networks of taste peptides based on global and local alignment algorithms. (A)** Global sequence similarity network constructed using the Needleman-Wunsch algorithm with spring layout algorithm for network visualization optimization. **(B)** Local sequence similarity network constructed using the Smith-Waterman algorithm with Kamada-Kawai layout algorithm to emphasize local sequence similarities. In both networks, nodes represent individual peptides with size proportional to sequence length, and node colors indicate different taste properties. Edge thickness corresponds to the degree of sequence similarity between peptides.


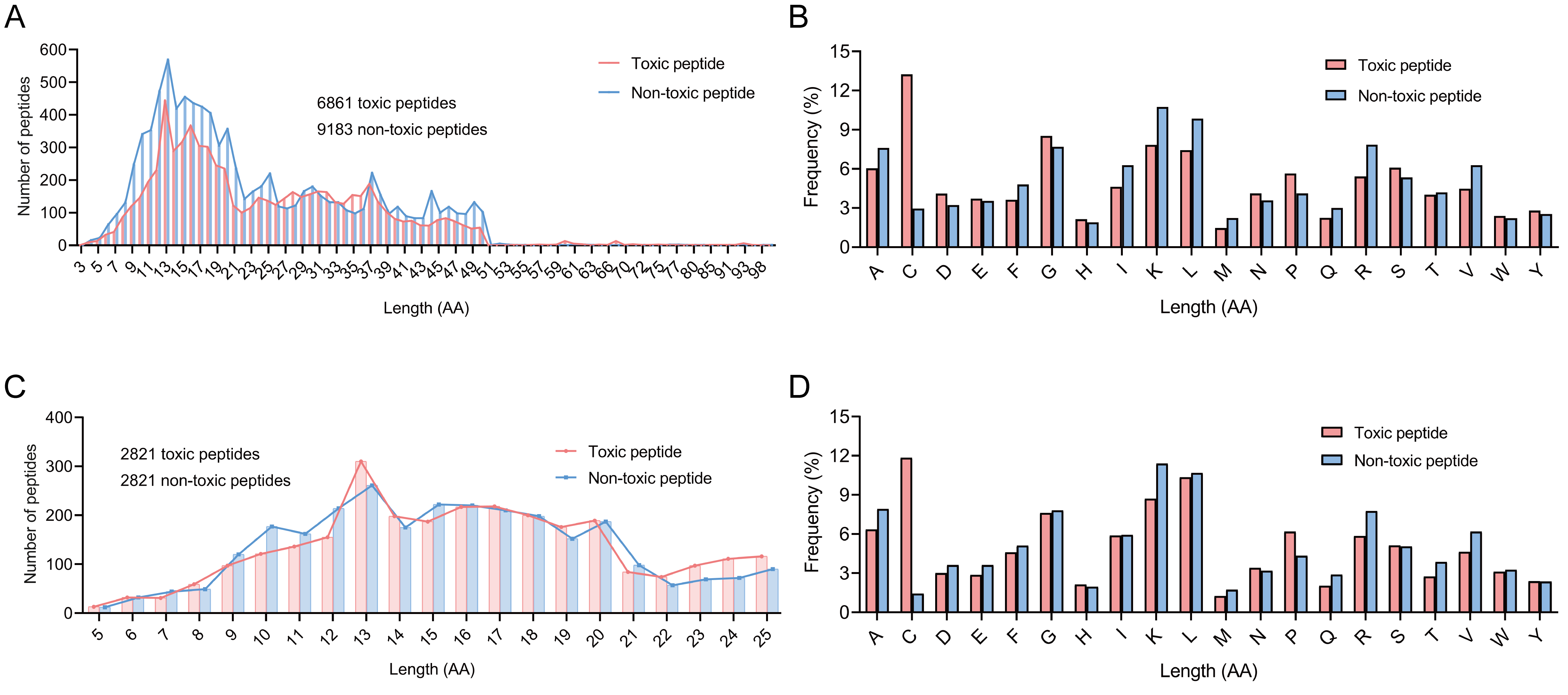


**Fig B. Length distribution and amino acid composition analysis of toxic and non-toxic peptides. (A)** Length distribution of the complete dataset comprising 6861 toxic peptides (red) and 9183 non-toxic peptides (blue). **(B)** Amino acid frequency distribution in the complete dataset. **(C)** Length distribution of the filtered dataset (≤25 AA) containing 2821 toxic peptides and 2821 length-matched non-toxic peptides. **(D)** Amino acid frequency distribution in the filtered dataset (≤25 AA).


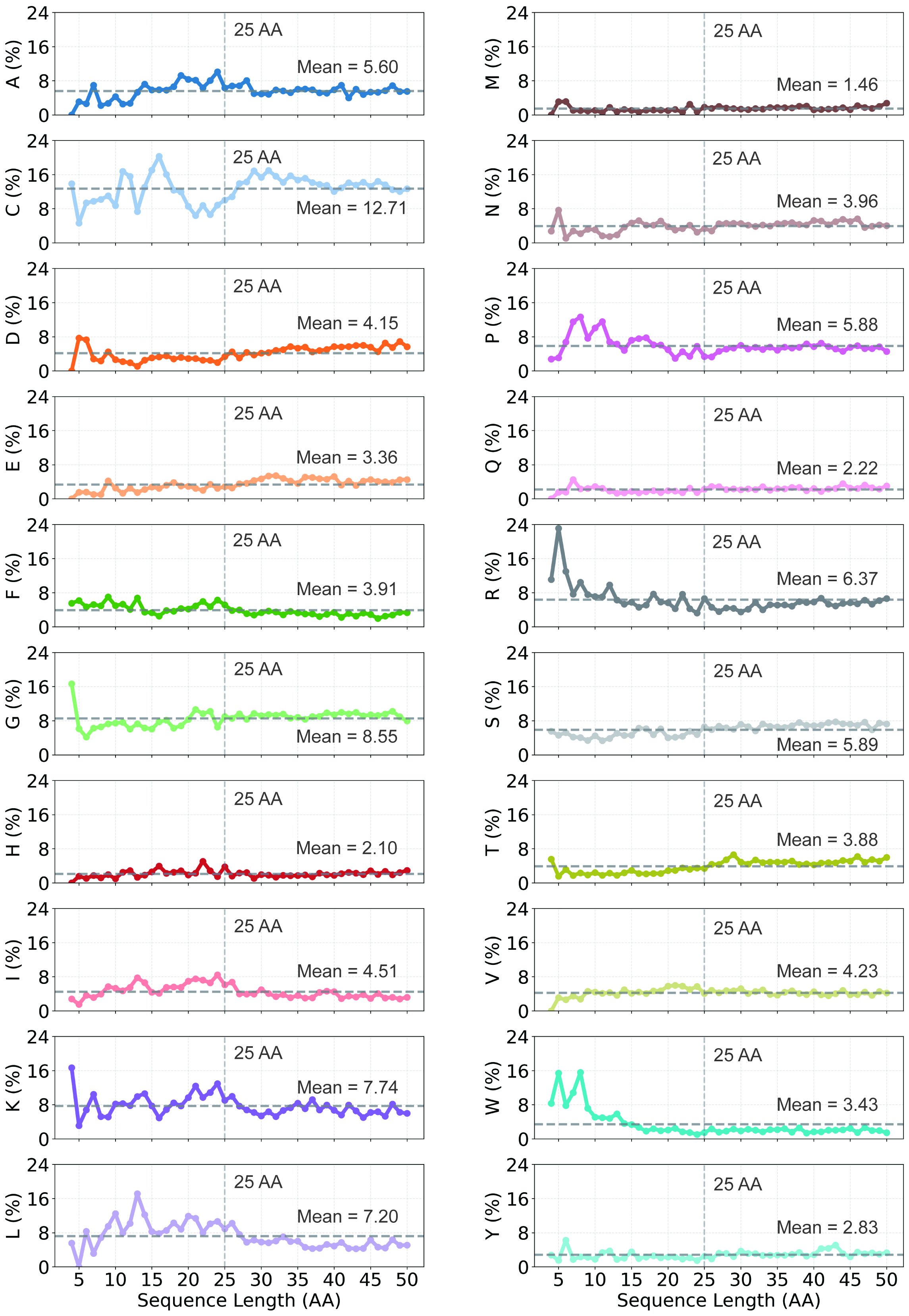


**Fig C. Length-specific amino acid frequency analysis of toxic peptides.** Frequency distribution analysis of amino acid residues across different sequence length groups in toxic peptides. Each subplot shows how a specific amino acid's frequency (%) varies among peptide sequence sets of different lengths (4-50 AA). The vertical dashed line at 25 AA indicates our sequence length threshold for model development. The mean frequency value is indicated for each residue.


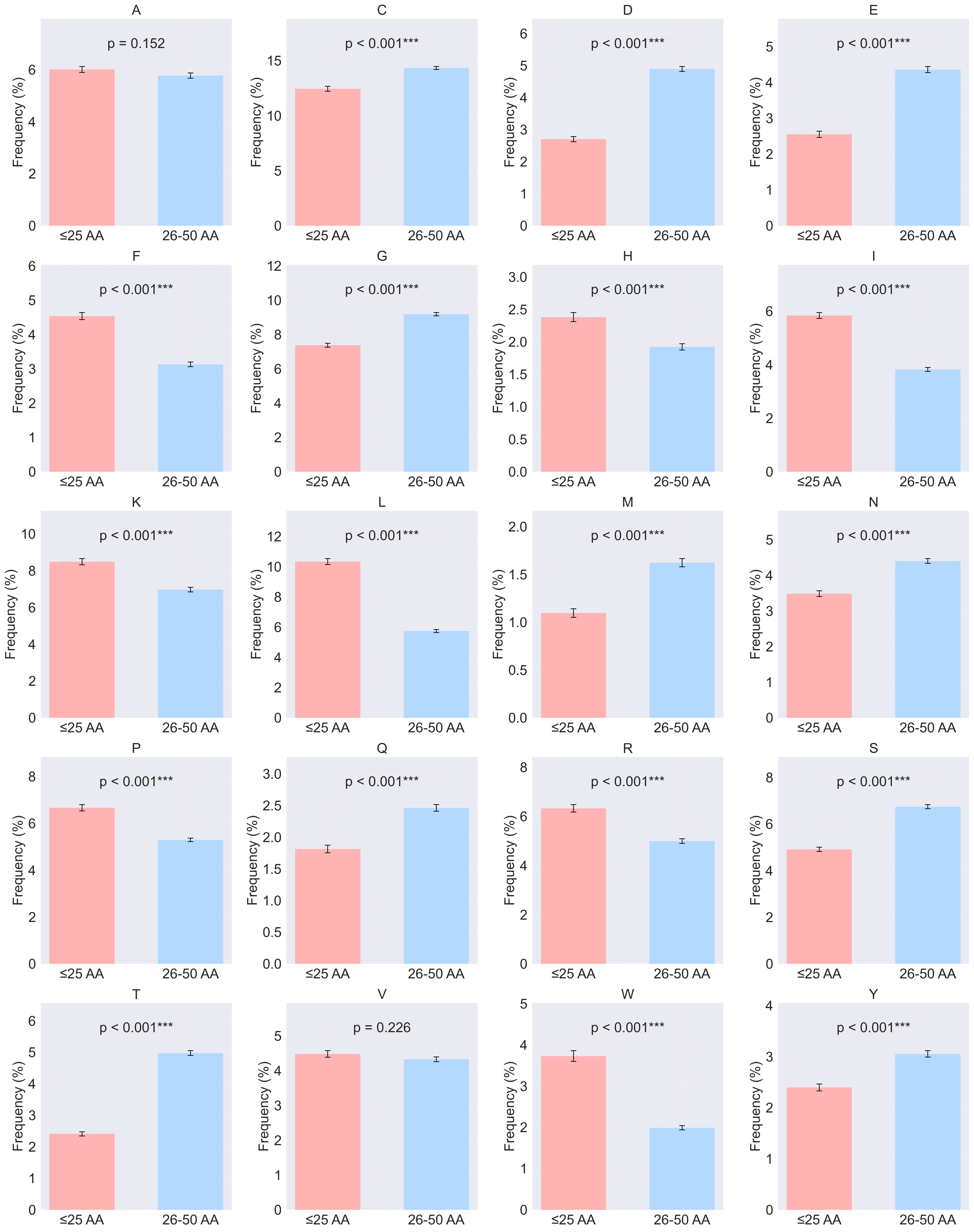


**Fig D. Comparative analysis of amino acid frequencies between shorter (≤25 AA) and longer (26-50 AA) toxic peptides.** Frequency distribution (%) of 20 amino acid residues in shorter (≤25 AA, pink) and longer (26-50 AA, blue) toxic peptide sequences. Error bars represent standard errors. Statistical significance levels are indicated (*p < 0.05, **p < 0.01, ***p < 0.001). Eighteen amino acids showed significant differences in their frequencies between the two length groups (p < 0.001), except for alanine (A, p = 0.152) and valine (V, p = 0.226), supporting the rationale for length-specific toxicity modeling.


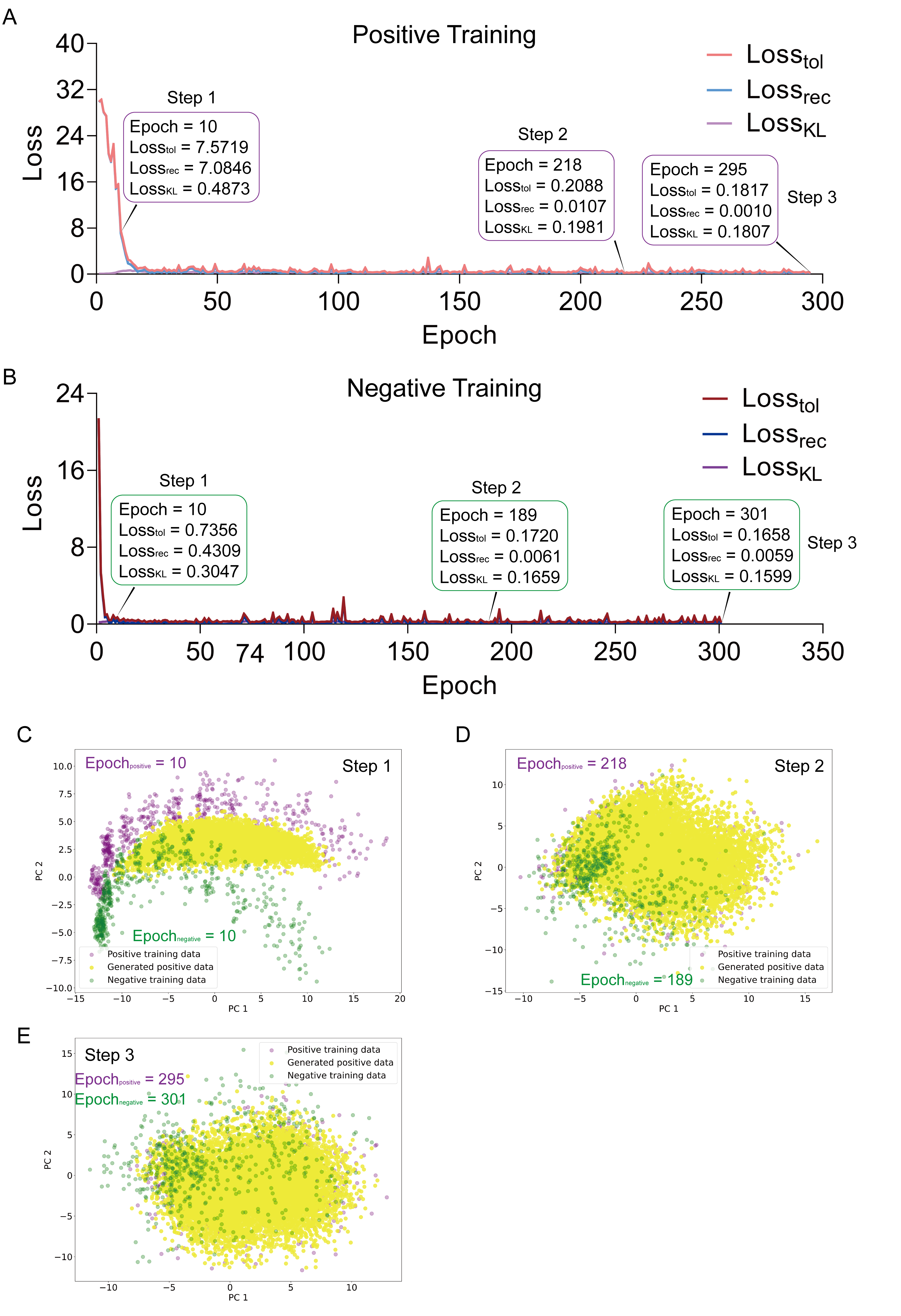


**Fig E. Training dynamics and latent space evolution of LA-VAE. (A, B)** Loss curves during model training for **(A)** positive and **(B)** negative data across 500 epochs. **(C-E)** Two-dimensional PCA visualization of the latent space distribution at different training stages. **(C)** Separation of positive (purple), negative (green), and generated positive (yellow) samples at Step 1 (epoch 10). **(D)** Intermediate stage showing increased overlap between generated and positive samples at Step 2. **(E)** Final convergence state demonstrating optimal distribution alignment at Step 3.


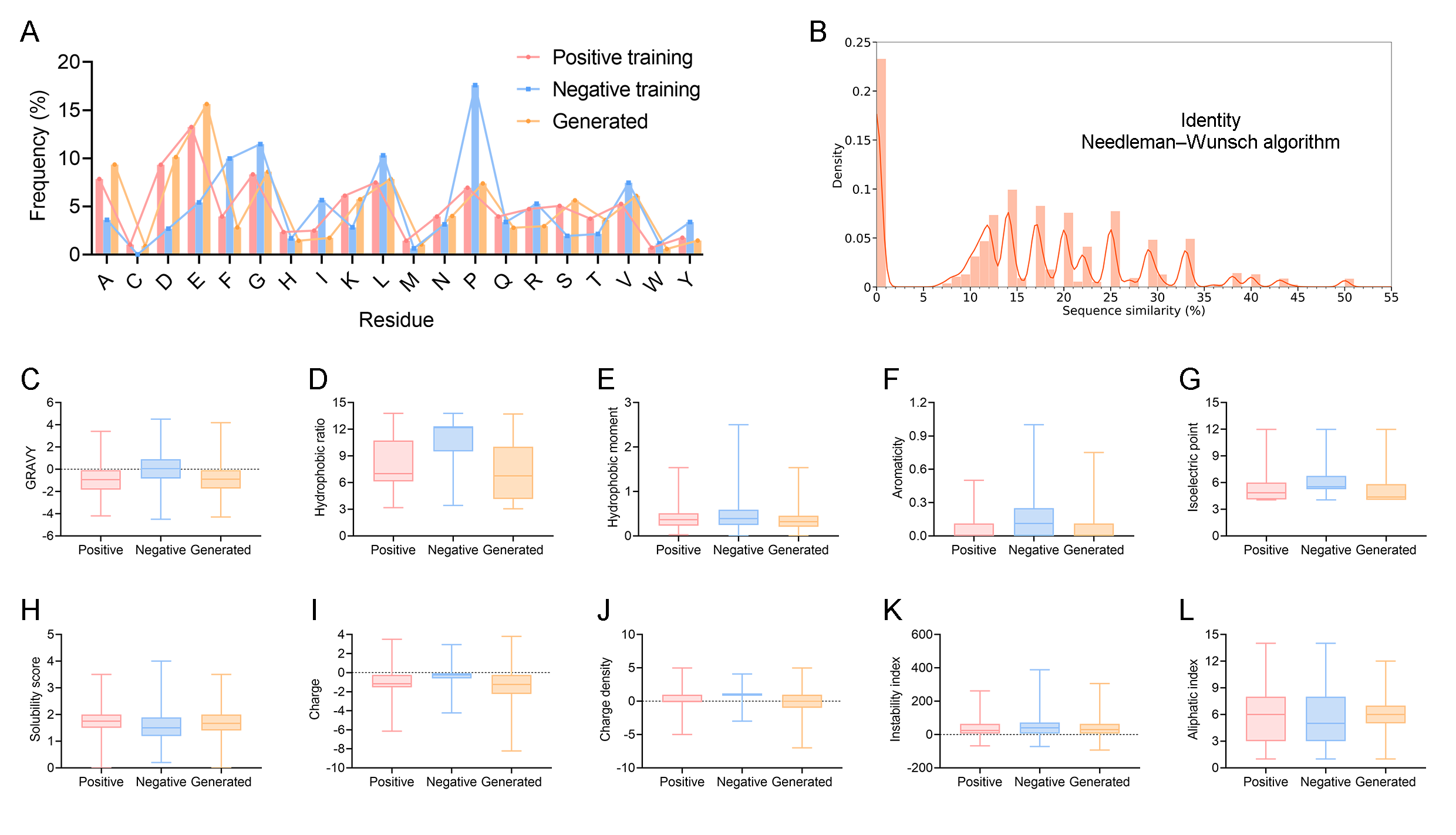


**Fig F. Sequence and physicochemical property analysis of 10000 TastePepAI-generated peptides. (A)** Amino acid residue frequency distribution (%) comparing positive training samples (red), negative training samples (blue), and generated sequences (orange). **(B)** Sequence similarity density distribution between generated peptides and positive training samples, calculated using the Needleman-Wunsch algorithm. The majority of generated sequences show less than 50% identity to training samples. **(C-L)** Box plots comparing physicochemical properties among positive training set (light red), negative set (light blue), and generated sequences (light orange). Box plots show median, quartiles, and whiskers (minimum to maximum).


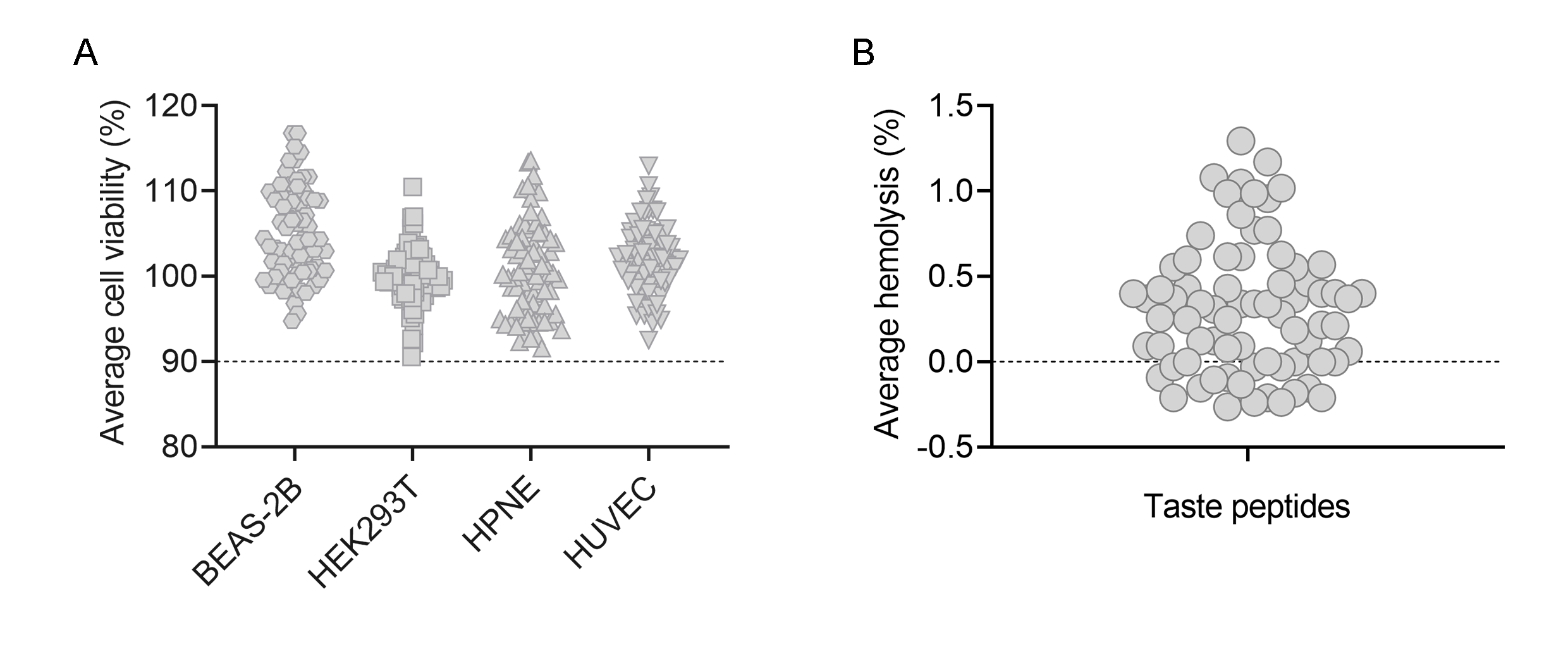


**Fig G. Safety evaluation of 73 peptides. (A)** Cell viability assessment of 73 peptides (100 μM) across four different cell lines: BEAS-2B, HEK293T, HPNE, and HUVEC. **(B)** Hemolysis rates of 73 peptides (100 μM) using mouse red blood cells.

**Table A. Electronic tongue analysis results of synthetic peptides at 1 mg/mL.**

| Peptide | Sour | Sweet | Bitter | Salty | Umami |
| --- | --- | --- | --- | --- | --- |
| TaPep1 | 9.07 | -3.13 | 3.53 | 13.72 | -3.64 |
| TaPep2 | 12.60 | -5.50 | 2.74 | 13.52 | -4.05 |
| TaPep3 | 12.21 | -6.65 | 2.95 | 13.20 | -4.90 |
| TaPep4 | 13.79 | -5.50 | 1.90 | 17.52 | -5.17 |
| TaPep5 | 12.61 | -5.63 | -0.10 | 14.26 | -5.17 |
| TaPep6 | 10.67 | -5.48 | 2.72 | 12.20 | -5.07 |
| TaPep7 | 11.92 | -6.18 | -0.45 | 9.19 | -5.11 |
| TaPep8 | 8.25 | -4.65 | 1.63 | 7.96 | -4.08 |
| TaPep9 | 8.17 | -4.48 | 2.75 | 12.49 | -2.73 |
| TaPep10 | 8.97 | -4.53 | -0.80 | 14.02 | -3.37 |
| TaPep11 | 9.08 | -5.23 | 3.24 | 6.18 | -2.91 |
| TaPep12 | 6.59 | -7.92 | 0.75 | 7.54 | -2.86 |
| TaPep13 | 12.34 | -4.65 | -0.78 | 15.30 | -4.92 |
| TaPep14 | 9.86 | -4.10 | 0.38 | 13.64 | -3.87 |
| TaPep15 | 8.97 | -3.92 | 4.11 | 2.97 | -3.63 |
| TaPep16 | 12.97 | -5.75 | 3.65 | 7.92 | -5.22 |
| TaPep17 | 14.54 | -5.15 | 3.52 | -0.53 | -5.94 |
| TaPep18 | 13.35 | -5.91 | 3.77 | 7.99 | -5.33 |
| TaPep19 | 10.91 | -4.08 | 3.19 | 8.70 | -4.34 |
| TaPep20 | 11.76 | -4.17 | 3.35 | 6.62 | -4.77 |
| TaPep21 | 12.04 | -10.15 | 3.66 | 7.62 | -5.14 |
| TaPep22 | 9.43 | -4.86 | 3.37 | 7.21 | -3.68 |
| TaPep23 | 11.00 | -4.58 | 3.47 | 6.69 | -4.39 |
| TaPep24 | 9.09 | -4.08 | 3.73 | 4.83 | -3.57 |
| TaPep25 | 7.84 | -3.53 | 3.50 | 5.51 | -3.13 |
| TaPep26 | 10.93 | -3.99 | 3.16 | 8.38 | -4.35 |
| TaPep27 | 14.83 | -6.25 | 3.79 | 8.93 | -6.01 |
| TaPep28 | 13.70 | -6.08 | 4.00 | 11.23 | -5.39 |
| TaPep29 | 10.60 | -5.49 | 3.20 | 9.53 | -4.08 |
| TaPep30 | 12.02 | -6.46 | 3.18 | 11.18 | -4.59 |
| TaPep31 | 12.70 | -5.90 | 2.76 | 12.57 | -4.82 |
| TaPep32 | 11.60 | -6.23 | 2.80 | 9.59 | -4.56 |
| TaPep33 | 10.86 | -5.32 | 3.28 | 10.92 | -0.93 |
| TaPep34 | 15.34 | -6.74 | 3.25 | 13.63 | -4.87 |
| TaPep35 | 12.93 | -7.23 | 2.77 | 10.87 | -4.82 |
| TaPep36 | 17.06 | -8.13 | 3.11 | 13.68 | -6.54 |
| TaPep37 | 12.49 | -5.26 | 3.28 | 15.18 | -5.88 |
| TaPep38 | 9.17 | -5.11 | 3.12 | 10.85 | -3.92 |
| TaPep39 | 15.66 | -7.82 | 2.99 | 12.96 | -5.89 |
| TaPep40 | 10.99 | -5.29 | 3.31 | 10.09 | -3.91 |
| TaPep41 | 9.16 | -8.94 | 3.91 | 2.54 | -3.94 |
| TaPep42 | 16.30 | -6.66 | 2.96 | 15.72 | -6.40 |
| TaPep43 | 12.46 | -3.45 | 4.00 | 7.60 | -5.11 |
| TaPep44 | 14.96 | -11.10 | 3.13 | 10.03 | -6.35 |
| TaPep45 | 12.61 | -4.05 | 4.03 | 9.35 | -5.11 |
| TaPep46 | 10.78 | -3.59 | 4.18 | 4.55 | -4.41 |
| TaPep47 | 10.97 | -5.60 | 3.37 | 9.73 | -4.54 |
| TaPep48 | 12.29 | -3.88 | 4.06 | 9.10 | -4.97 |
| TaPep49 | 8.30 | -3.73 | 2.53 | 11.00 | -3.34 |
| TaPep50 | 9.63 | -3.07 | 4.27 | 3.54 | -3.90 |
| TaPep51 | 5.93 | -1.16 | 7.16 | 8.48 | -2.27 |
| TaPep52 | 5.72 | -1.51 | 4.10 | 3.13 | -2.08 |
| TaPep53 | 8.92 | -4.92 | 2.74 | 13.59 | -3.47 |
| TaPep54 | 8.51 | -6.29 | 2.50 | 10.59 | -3.47 |
| TaPep55 | 11.12 | -7.81 | 2.75 | 11.30 | -4.53 |
| TaPep56 | 9.59 | -7.49 | 3.33 | 6.57 | -4.04 |
| TaPep57 | 12.57 | -7.93 | 3.08 | 9.94 | -5.02 |
| TaPep58 | 10.37 | -6.69 | 3.43 | 10.46 | -4.23 |
| TaPep59 | 15.44 | -7.32 | 1.90 | 15.85 | -6.02 |
| TaPep60 | 12.51 | -9.69 | 3.00 | 9.00 | -5.30 |
| TaPep61 | 6.60 | -2.71 | 3.33 | 3.55 | -2.71 |
| TaPep62 | 11.86 | -4.13 | 3.70 | 8.50 | -4.77 |
| TaPep63 | 15.39 | -5.33 | 4.12 | 10.16 | -6.21 |
| TaPep64 | 9.90 | -4.54 | 3.04 | 10.50 | -4.02 |
| TaPep65 | 10.48 | -3.79 | 4.02 | 11.34 | -4.04 |
| TaPep66 | 8.49 | -3.10 | 4.03 | 4.47 | -3.37 |
| TaPep67 | 10.40 | -3.54 | 4.01 | 9.82 | -4.10 |
| TaPep68 | 12.33 | -4.04 | 3.94 | 12.33 | -4.79 |
| TaPep69 | 11.54 | -4.24 | 3.75 | 9.06 | -4.62 |
| TaPep70 | 10.04 | -3.62 | 3.77 | 7.83 | -4.00 |
| TaPep71 | 14.75 | -5.65 | 4.11 | 9.59 | -5.94 |
| TaPep72 | 11.06 | -5.31 | 3.83 | 7.83 | -4.42 |
| TaPep73 | 13.38 | -5.09 | 4.33 | 8.78 | -5.41 |

**Table B. Electronic tongue analysis results of synthetic peptides at 0.1 mg/mL.**

| Peptide | Sour | Sweet | Bitter | Salty | Umami |
| --- | --- | --- | --- | --- | --- |
| TaPep1 | -4.53 | 6.22 | 10.96 | -19.14 | 2.15 |
| TaPep2 | -2.30 | 6.09 | 7.88 | -19.03 | 1.21 |
| TaPep3 | -4.64 | 7.95 | 7.84 | -20.13 | 2.16 |
| TaPep4 | -2.92 | 4.21 | 7.74 | -16.43 | 1.35 |
| TaPep5 | -4.04 | 6.12 | 7.90 | -18.14 | 1.93 |
| TaPep6 | -4.43 | 8.22 | 7.90 | -19.24 | 1.99 |
| TaPep7 | -2.86 | 6.98 | 7.41 | -21.22 | 1.28 |
| TaPep8 | -5.63 | 9.31 | 7.50 | -22.00 | 2.66 |
| TaPep9 | -6.31 | 8.92 | 7.95 | -18.92 | 2.67 |
| TaPep10 | -8.60 | 11.07 | 6.97 | -20.44 | 3.61 |
| TaPep11 | -5.95 | 9.69 | 7.10 | -21.79 | 2.75 |
| TaPep12 | -6.74 | 7.49 | 8.03 | -17.83 | 3.01 |
| TaPep13 | -3.77 | 3.50 | 7.46 | -15.97 | 1.81 |
| TaPep14 | -6.58 | 7.88 | 7.83 | -18.09 | 3.07 |
| TaPep15 | -7.15 | 9.72 | 6.27 | -23.91 | 3.17 |
| TaPep16 | -2.22 | 4.77 | 7.29 | -21.97 | 1.15 |
| TaPep17 | -2.22 | 5.23 | 7.25 | -21.65 | 1.17 |
| TaPep18 | -3.88 | 5.97 | 6.92 | -22.88 | 1.84 |
| TaPep19 | -4.23 | 6.20 | 7.19 | -21.50 | 2.00 |
| TaPep20 | -2.48 | 5.13 | 7.24 | -21.79 | 1.29 |
| TaPep21 | -1.59 | 5.53 | 7.83 | -19.46 | 0.99 |
| TaPep22 | -5.65 | 7.18 | 6.91 | -22.87 | 2.56 |
| TaPep23 | -3.15 | 5.26 | 7.18 | -22.67 | 1.53 |
| TaPep24 | -4.17 | 6.22 | 7.18 | -22.67 | 1.53 |
| TaPep25 | -6.12 | 8.12 | 7.01 | -23.02 | 1.53 |
| TaPep26 | -3.53 | 5.51 | 7.26 | -21.44 | 1.71 |
| TaPep27 | -0.70 | 3.39 | 7.42 | -22.39 | 0.54 |
| TaPep28 | -2.05 | 3.25 | 7.58 | -21.08 | 1.08 |
| TaPep29 | -3.61 | 7.74 | 7.69 | -20.79 | 1.81 |
| TaPep30 | -3.43 | 5.66 | 7.53 | -20.28 | 1.69 |
| TaPep31 | -3.49 | 8.41 | 7.84 | -19.57 | 1.69 |
| TaPep32 | -3.82 | 8.60 | 7.51 | -20.95 | 1.89 |
| TaPep33 | -4.09 | 8.49 | 7.77 | -19.69 | 1.93 |
| TaPep34 | -0.52 | 5.08 | 7.62 | -17.51 | 0.43 |
| TaPep35 | -3.84 | 8.65 | 7.64 | -20.10 | 1.93 |
| TaPep36 | -0.61 | 5.62 | 7.85 | -18.92 | 0.55 |
| TaPep37 | -4.03 | 4.01 | 7.34 | -13.82 | 1.93 |
| TaPep38 | -5.92 | 7.68 | 7.67 | -19.89 | 2.66 |
| TaPep39 | -1.65 | 6.53 | 7.69 | -18.68 | 0.99 |
| TaPep40 | -5.48 | 10.64 | 7.48 | -19.59 | 2.37 |
| TaPep41 | -3.84 | 8.41 | 7.34 | -20.79 | 1.88 |
| TaPep42 | -0.03 | 3.04 | 7.41 | -15.26 | 0.30 |
| TaPep43 | -1.87 | 5.59 | 7.83 | -20.35 | 1.07 |
| TaPep44 | -0.62 | 3.94 | 7.74 | -19.30 | 0.51 |
| TaPep45 | -1.77 | 4.54 | 7.77 | -19.03 | 0.96 |
| TaPep46 | -3.21 | 6.32 | 7.55 | -21.33 | 1.60 |
| TaPep47 | -4.89 | 6.95 | 8.00 | -19.15 | 2.32 |
| TaPep48 | -2.20 | 5.24 | 7.92 | -19.00 | 1.19 |
| TaPep49 | -7.10 | 7.69 | 7.26 | -15.59 | 0.96 |
| TaPep50 | -4.45 | 7.42 | 7.18 | -22.53 | 2.11 |
| TaPep51 | -8.80 | 12.64 | 9.91 | -19.53 | 3.88 |
| TaPep52 | -6.24 | 9.54 | 7.49 | -20.56 | 2.84 |
| TaPep53 | -6.36 | 9.02 | 7.88 | -19.95 | 2.86 |
| TaPep54 | -7.11 | 9.28 | 7.63 | -20.20 | 3.06 |
| TaPep55 | -2.34 | 5.21 | 7.76 | -19.80 | 1.27 |
| TaPep56 | -4.96 | 7.84 | 7.18 | -21.19 | 2.28 |
| TaPep57 | -3.54 | 6.75 | 7.29 | -21.64 | 1.71 |
| TaPep58 | -4.24 | 7.50 | 7.49 | -21.68 | 2.07 |
| TaPep59 | -3.89 | 6.34 | 7.83 | -19.53 | 1.85 |
| TaPep60 | -2.65 | 5.70 | 7.64 | -20.71 | 1.33 |
| TaPep61 | -7.21 | 8.89 | 6.59 | -23.88 | 3.14 |
| TaPep62 | -2.87 | 6.38 | 7.43 | -20.91 | 1.43 |
| TaPep63 | -2.72 | 5.62 | 7.27 | -20.42 | 1.41 |
| TaPep64 | -4.25 | 4.85 | 7.39 | -21.79 | 1.91 |
| TaPep65 | -3.95 | 2.66 | 7.27 | -17.33 | 1.85 |
| TaPep66 | -3.77 | 6.93 | 7.14 | -21.50 | 1.83 |
| TaPep67 | -4.19 | 6.41 | 7.39 | -19.59 | 1.99 |
| TaPep68 | -1.26 | 3.06 | 7.39 | -15.75 | 0.75 |
| TaPep69 | -2.38 | 5.65 | 7.32 | -19.29 | 1.23 |
| TaPep70 | -3.33 | 6.55 | 7.38 | -20.29 | 1.63 |
| TaPep71 | -2.77 | 5.63 | 7.30 | -20.93 | 1.39 |
| TaPep72 | -4.62 | 7.58 | 7.25 | -19.66 | 2.15 |
| TaPep73 | -1.22 | 4.76 | 7.64 | -19.69 | 0.77 |
