# Supplementary material for: TastepepAI: An artificial intelligence platform for taste peptide de novo design
Source: PLoS Comput Biol. 2025 Oct 16;21(10):e1013602. doi: 10.1371/journal.pcbi.1013602 (PMC12543283; doi:10.1371/journal.pcbi.1013602)
Supplement: Attachment — Submitted filename: response.docx [file pcbi.1013602.s007.docx]

PCOMPBIOL-D-25-01329
TastepepAI:An artificial intelligence platform for taste peptide de novo design
PLOS Computational Biology

Dear Dr. Wang,

Thank you for submitting your manuscript to PLOS Computational Biology. As with all papers, your manuscript was reviewed by members of the editorial board. Based on our assessment, we have decided that the work does not meet our criteria for publication and will therefore be rejected. If external reviews were secured, reviewers' comments will be included at the bottom of this email.

We are sorry that we cannot be more positive on this occasion. We very much appreciate your wish to present your work in one of PLOS's Open Access publications. Thank you for your support, and we hope that you will consider PLOS Computational Biology for other submissions in the future.

Yours sincerely,

Arne Elofsson
Section Editor
PLOS Computational Biology

Arne Elofsson
Section Editor
PLOS Computational Biology

**Additional Editor Comments (if provided):**

This paper could be suitable for ploscb as a methods paper. However, from what I can see the code is not available with an open access license i which is a requirement for such papers. Therefore, you would have to make it available and resubmit this manuscript before we can handle it.

Response:

We have uploaded the source code at https://github.com/leleshidawang/TastepepAI.
