# Supplementary material for: TastepepAI: An artificial intelligence platform for taste peptide de novo design
Source: PLoS Comput Biol. 2025 Oct 16;21(10):e1013602. doi: 10.1371/journal.pcbi.1013602 (PMC12543283; doi:10.1371/journal.pcbi.1013602)
Supplement: Attachment — Submitted filename: Response_to_the_reviewers_0919.docx [file pcbi.1013602.s008.docx]

Reviewer #1: Yue et al. developed an artificial intelligence platform for designing taste peptides. This manuscript addresses a topic that may be of interest to ploscb as a methods paper. This work provides an artificial intelligence tool for generating target peptides with the desired taste profiles. This approach allows for a reduction in time and resources, thus enabling broader application in the food industry. Although generally well-structured and presented, there are two points that could be further clarified to support this work.

In the results, section 2.2 LA-VAE: a loss-supervised adaptive variational autoencoder with contrastive learning for controlled taste peptide generation paragraph, it's not clear what has been done:

‘To meet the demands for precise control of specific taste properties (e.g., bitter taste elimination40,53) in practical applications, we incorporated a contrastive learning-based properties, enabling bilateral partitioning of training data: sequences with target taste avoidance mechanism into the LA-VAE architecture (Figure 3B) This mechanism allows users to explicitly specify desired (preferred) and avoided (aversive) taste features constitute the positive set, while those with avoided features form the negative set. Through parallel training on these complementary datasets, LA-VAE establishes a structured contrastive representation framework in the latent space.‘

More specifically, what is meant by "bilateral partitioning of the training data"? Furthermore, does this "partitioning" reduce the initial dataset and thus the model training? How does this partitioning ultimately impact decision-making or predictions?

Thank you for raising this important technical clarification question. Drawing upon the methodological sections of our paper, we provide a detailed explanation of the theoretical foundation and implementation mechanism of "bilateral partitioning of training data" in LA-VAE.

1. Theoretical background:

Our "bilateral partitioning" strategy stems from a significant improvement in our taste annotation system. As detailed in method 4.1.1, we initially employed a five-digit binary system (>abcde) to represent the presence (1) or absence (0) of five basic tastes, but this approach had limitations. Considering the complexity of taste perception, experimental condition variations, and the scientific fact that "the absence of a reported taste in existing studies or its non-detection through specific experimental procedures should not definitively indicate its non-existence," we developed a more precise annotation system using 'x' to mark unconfirmed taste properties, indicating "uncertainty."

2. Interactive taste feature definition and activation mechanism:

As detailed in method 4.2.1, users define target taste characteristics through five-digit codes ('1' indicates desired features, '0' represents features to avoid, 'x' denotes no specific requirement). Crucially: when user input patterns include avoidance features (marked as '0'), the system automatically activates contrastive learning strategies and the bilateral partitioning mechanism.

3. Specific meaning of "bilateral partitioning":

"Bilateral partitioning" refers to the system's intelligent division of the complete training dataset into two functionally complementary subsets based on user-defined taste preference patterns:

- Positive set: sequences matching user-desired taste feature patterns
- Negative set: sequences containing taste features that users wish to avoid

Illustrative example: when a user inputs '>x10xx' (designing a peptide with sweet taste while avoiding bitter taste):

Positive set: all sequences annotated with patterns matching '>x10xx', where positions 1, 4, and 5 can be 0, 1, or x, but position 2 must be 1, and position 3 must be 0

Negative set: all sequences containing bitter taste (position 3='1') in their annotations

The specific implementation mechanism can be found in our publicly available github code 02_LA-VAE.py. As shown below:

4. Impact on dataset size:

This partitioning approach does not reduce the total amount of data available for training; rather, it enables more comprehensive data utilization. In "contrastive learning" mode, the system fully utilizes both positive and negative samples for training, effectively increasing the utilization of effective training data.

5. Impact on decision-making and predictions:

The bilateral partitioning mechanism significantly enhances model performance through the following approaches (Figure 3, Figure S5C-E, Figure S6):

- Latent space contrastive learning: positive and negative samples form contrastive distributions in the latent space, helping the model learn to distinguish between desired and avoided features
- Distance-based screening: generated sequences undergo k-nearest neighbor distance evaluation (k=5), calculating average Euclidean distances to positive and negative samples
- Dual-constraint optimization: only generated sequences that simultaneously satisfy "proximity to positive samples" and "distance from negative samples" are selected as final outputs

This bilateral partitioning strategy represents a core innovation in TastePepAI's precise taste control capabilities, particularly suitable for practical applications in the food industry requiring "enhancement of certain tastes while suppressing others."

We hope this detailed technical explanation addresses your concerns. All relevant implementation code is publicly available on github: <https://github.com/leleshidawang/TastepepAI>

The changes have been made as follow,

“To meet the demands for precise control of specific taste properties (e.g., bitter taste elimination^40,53^) in practical applications, we incorporated a contrastive learning-based taste avoidance mechanism into the LA-VAE architecture (Figure 3B). Our contrastive approach differs from traditional contrastive learning methods that use explicit contrastive loss functions. Instead, it implements an implicit contrastive mechanism through bilateral data partitioning and latent space distance evaluation. This mechanism allows users to explicitly specify desired (preferred) and avoided (aversive) taste properties, enabling bilateral partitioning of training data: sequences with target taste features constitute the positive set, while those with avoided features form the negative set. Through parallel training on these complementary datasets, LA-VAE establishes a structured contrastive representation framework in the latent space.”

In the results section, 2.4. Design and validation of safe taste peptides via TastePepAI paragraph:

If the training data with "currently no bitterness" were removed, would this have a negative or positive impact on the model's learning?

Thank you for raising this important data quality question. To quantitatively assess the potential impact of removing "currently no bitterness" sequences (i.e., sequences with position 3 marked as 'x' indicating no bitter taste) on model training, we conducted a detailed statistical analysis of our training dataset (Input_taste_peptides.fasta, available for download at https://github.com/leleshidawang/TastepepAI). The results reveal that removing these sequences would have severe negative impacts on model learning, as detailed below:

1. Substantial reduction in training data

Our statistical analysis shows that non-bitter sequences account for 52.2% (590/1131) of the total dataset. Removing these sequences would reduce the training data from 1131 to only 541 samples, representing a loss of more than half the data. This massive reduction in training data would severely compromise the training effectiveness of deep learning models, particularly for VAE architectures that require substantial amounts of data to learn complex sequence-function relationships.

2. Catastrophic loss of taste combination diversity

More critically, removing non-bitter sequences would result in a catastrophic loss of taste combination diversity:

The original 28 distinct taste combination types would be reduced to only 13 types, representing a loss rate of 53.6%

The following 15 complete taste combination patterns would entirely disappear:

- 11x11 (sweet + salty + umami, 5 sequences)
- 11x1x (sweet + salty + sour, 1 sequence)
- 11xx1 (sweet + salty + umami, 25 sequences)
- 11xxx (sweet + salty, 3 sequences)
- 1xx11 (sweet + sour + umami, 20 sequences)
- 1xx1x (sweet + sour, 2 sequences)
- 1xxx1 (sweet + umami, 80 sequences)
- 1xxxx (pure sweet, 17 sequences)
- x1x11 (salty + sour + umami, 8 sequences)
- x1x1x (salty + sour, 1 sequence)
- x1xx1 (salty + umami, 71 sequences)
- x1xxx (pure salty, 26 sequences)
- xxx11 (sour + umami, 18 sequences)
- xxx1x (pure sour, 57 sequences)
- xxxx1 (pure umami, 256 sequences)

Among these, xxxx1 (pure umami) represents the second-largest taste category in the dataset, while 1xxx1 (sweet + umami) and x1xx1 (salty + umami) are important dual-taste combination patterns.

3. Specific impacts on model learning capability

Based on the above analysis, removing non-bitter sequences would result in:

- Severe positive sample deficiency: Available training positive samples would decrease by 52.2%, with some rare taste combination patterns having their sample counts reduced from dozens to zero
- Severely compromised model generalization: Loss of 15 complete taste combination types means the model would entirely lose the opportunity to learn these important taste patterns and would be unable to generate corresponding functional peptides
- Overly conservative generation strategy: Training based solely on the remaining 13 taste combinations would make the model overly restrictive, significantly reducing the diversity and innovation of generated sequences

4. Discussion

Given the severe negative consequences of removing "currently no bitterness" sequences, we believe that simple data deletion strategies are unfeasible. Such crude treatment would severely damage TastePepAI's learning capability and application value.

As an alternative, we propose adopting an experimental validation-based data correction strategy:

- Batch wet-lab validation: First, randomly select a representative batch of samples from sequences marked as "currently no bitterness" for wet-lab validation to determine their true taste properties
- Active learning strategy: Based on preliminary validation results, employ active learning methods to identify training samples most likely to contain annotation errors and prioritize them for experimental verification
- Progressive data correction: Gradually correct training data annotations based on experimental validation results, rather than removing large amounts of data at once
- Uncertainty modeling: Introduce annotation uncertainty weights during model training to appropriately modulate the influence of suspicious annotations

This experimental validation and active learning-based data quality improvement strategy would maintain the integrity and diversity of training data while gradually improving annotation accuracy, thereby genuinely enhancing TastePepAI's performance.

We want to emphasize that your question provides excellent insights and significant inspiration for our future work. The wet-lab validation of training datasets will be our primary consideration for upgrading TastePepAI's performance in future iterations. This approach represents a more scientifically rigorous and practically valuable direction for improving computational taste peptide design models.

‘This limited bitterness suppression might be attributed to residual impurities from peptide synthesis (e.g., trifluoroacetic acid, sodium acetate) potentially interfering with sensory evaluation10,56,57, coupled with possible taste evaluation blind spots in training data labeled as ‘currently without bitterness’58,59. Consequently, accurate assessment of bitterness suppression requires further validation.’

How do the authors intend to proceed in validating the suppression of bitterness?

Thank you for your attention to our validation methodology. Although our research primarily focuses on computational biology, we fully recognize the importance of wet-lab experimental validation for confirming whether model-generated peptides truly reduce or eliminate bitterness. We plan to address this critical question through the following three-tier experimental validation framework:

To eliminate the interference of synthetic impurities on taste evaluation, we plan to improve peptide acquisition methods. Specifically, we will attempt to employ recombinant expression and purification as an alternative to traditional chemical synthesis methods, thereby avoiding the influence of chemical synthesis byproducts such as trifluoroacetic acid (TFA) and sodium acetate. Simultaneously, we will establish more stringent peptide purification protocols, including multi-step chromatographic purification and strict quality control standards, to ensure the acquisition of target peptide sequences with higher purity and fewer impurities.

Based on high-purity peptide samples, we plan to seek collaborative laboratories with appropriate experimental capabilities to conduct in vitro receptor binding affinity assays. The specific experiments will determine the binding affinity between designed peptides and the TAS2R bitter taste receptor family. Through quantitative analysis of peptide-receptor interaction strength, we will validate from a molecular mechanistic perspective whether our designed peptides indeed possess the biological basis for reducing bitter taste perception.

The most crucial validation component is establishing a standardized human sensory evaluation system. After obtaining relevant ethical approvals, we plan to establish a professionally trained taste evaluation team and employ standardized sensory evaluation protocols to conduct systematic human trials on the generated taste peptides. The experimental design will include elements such as double-blind controls, multi-concentration gradient testing, and professional sensory panel assessments to ensure the objectivity and reliability of evaluation results.

This multi-tier validation framework will comprehensively validate TastePepAI's actual effectiveness in bitterness control from three dimensions: material purity, molecular mechanisms, and human sensory evaluation. Although these validation experiments present certain challenges for our team in the short term, we believe this represents a necessary step for advancing computational biology research toward practical applications. It is important to emphasize that the primary significance of our current study lies in the development of TastePepAI, a novel computational framework that opens new possibilities for rational taste peptide design. We assure you that our experimental research efforts will continue unabated, and we will prioritize these validation efforts in our future research plans while actively seeking collaborative opportunities in related fields to provide solid experimental support for our computational models.

Reviewer #2: The manuscript introduces TastePepAI, an innovative AI framework for customized taste peptide design and safety assessment. The integration of LA-VAE with a toxicity prediction module represents a promising advance in computational peptide engineering and addresses a timely challenge in food science. However, the work suffers from insufficient methodological detail and limited validation.

1. During LA-VAE training, amino acid sequences are reconstructed via an autoencoder to obtain latent representations. However, it remains unclear which two distributions are compared in the KL divergence term. The paper mentions contrastive learning, but does not specify how the contrastive loss is calculated. Please clearly define all loss functions used, together with their weighting scheme in the total loss.

In our LA-VAE architecture, the KL divergence term compares the encoder-learned posterior distribution q(z|x) with the prior distribution p(z). Specifically, the posterior distribution q(z|x) is a parameterized Gaussian distribution defined by the mean vector z_mean and log-variance vector z_log_var output by the encoder, while the prior distribution p(z) is a standard multivariate Gaussian distribution N(0, I), where I is the identity matrix. This KL divergence regularization ensures that the learned latent representations follow a standard normal distribution, enabling effective sampling and interpolation in the latent space for peptide generation.

Code implementation (02_LA-VAE.py, available from github):

We have added clarification regarding the KL divergence term in section 4.2.2:

“Specifically, in the LA-VAE architecture, the KL divergence term compares the encoder-learned posterior distribution q(z|x) with the prior distribution p(z), where the posterior distribution q(z|x) is a parameterized Gaussian distribution defined by the mean vector z_mean and log-variance vector z_log_var output by the encoder, while the prior distribution p(z) is a standard multivariate Gaussian distribution N(0, I).”

The contrastive learning mentioned in our LA-VAE approach differs from traditional contrastive learning methods that use explicit contrastive loss functions. Instead, our method implements an implicit contrastive mechanism through bilateral data partitioning and latent space distance evaluation. To clarify our contrastive learning approach for readers, we have added the following clarification in the second paragraph of section 2.2:

“Our contrastive approach differs from traditional contrastive learning methods that use explicit contrastive loss functions. Instead, it implements an implicit contrastive mechanism through bilateral data partitioning and latent space distance evaluation.”

Additionally, the code implementation of contrastive learning in LA-VAE operates through the following mechanisms:

(1) When users specify taste patterns containing ‘0’ (indicating tastes to avoid), the system automatically partitions training data into positive and negative sets:

(2) Both positive and negative datasets are separately processed through the VAE architecture:

(3) The contrastive mechanism is implemented through k-nearest neighbor (k=5) bilateral distance evaluation in the latent space:

(4) The final sequence selection employs a differential ranking system that retains the top 25% of candidates with the smallest differential values, ensuring sequences demonstrate both positive sample affinity and negative sample repulsion:

This approach achieves contrastive learning effects without introducing explicit contrastive loss terms in the training objective, instead utilizing post-generation filtering based on latent space topology to ensure precise taste feature control.

Upon careful review of the entire manuscript, we identified that the loss function definitions exhibited inconsistent terminology and lacked clear detailed mathematical explanations. The paper involves three loss functions: the total loss function (${Loss}_{tol}$), reconstruction loss (${Loss}_{rec}$), and KL divergence loss (${Loss}_{KL}$).

We have added clear mathematical formula definitions for all loss functions in section 4.2.2, with explicit specification of the weighting scheme for each loss component. The complete mathematical formulations are presented as follows:

“The corresponding loss functions are mathematically formulated as follows. The total loss function of LA-VAE comprises two main components with equal weighting:

$${Loss}_{tol}={Loss}_{rec}+{Loss}_{KL}$$

The reconstruction loss employs binary cross-entropy with dimensional scaling to balance the contribution relative to the KL divergence term:

$${Loss}_{rec}=\frac{1}{N}\sum_{i=1}^{N} \left[ -\sum_{j=1}^{14} \sum_{k=1}^{21} \left[ x_{i,j,k}\log\left( \hat{x}_{i,j,k} \right)+\left( 1-x_{i,j,k} \right)\log\left( 1-\hat{x}_{i,j,k} \right) \right] \right]\times294$$

where $N$ is the batch size, $x_{i,j,k}$ represents the one-hot encoded input for the $i$-th sample at position $j$ and amino acid $k$, $\hat{x}_{i,j,k}$ is the corresponding reconstructed output, and the scaling factor $294=14\times21$ ensures balanced optimization between reconstruction and regularization terms.

The KL divergence loss follows the standard VAE formulation:

$${Loss}_{KL}=-\frac{0.5}{N}\sum_{i=1}^{N} \sum_{d=1}^{2000} \left[ 1+\log\sigma_{i,d}^{2}-\left( \mu_{i,d} \right)^{2}-\exp\left( \log\sigma_{i,d}^{2} \right) \right]$$

where $\mu_{i,d}$ and $\log\sigma_{i,d}^{2}$ are the mean and log-variance outputs from the encoder for the $d$-th latent dimension of the $i$-th sample, respectively. Both loss components utilize equal weighting (1:1 ratio) to ensure balanced optimization between reconstruction fidelity and latent space regularization.”

Additionally, we conducted a comprehensive review of the entire manuscript to ensure complete unification of the terminology for all three loss functions throughout the paper. The standardized nomenclature is now consistently used as follows: “total loss” (${Loss}_{tol}$), “reconstruction loss” (${Loss}_{rec}$), and “KL divergence loss” (${Loss}_{KL}$).

2. The inference stage is insufficiently explained. What is the input during inference? If it is only the desired taste profile (e.g., a character string of taste attributes), how are the corresponding candidate peptide sequences generated from this input?

Thank you for the reviewer's attention. We have revised section 4.2.1 to clarify the inference stage data input process. The modified content in section 4.2.1 now states:

“The system offers two operational modes: ‘Single Pattern Mode’ and ‘Multiple Pattern Mode’. In ‘Single Pattern Mode’, users input a single taste feature code (e.g., ‘>x1x0x’), and the system filters sequences from the database where the sweet taste position is marked as ‘1’ to form the positive sample set, while simultaneously collecting sequences where the salty taste position is marked as ‘1’ as the negative sample set, thereby achieving precise contrastive learning. In ‘Multiple Pattern Mode’, users can simultaneously input multiple patterns (e.g., ‘>x10xx,xx0x1’), and the system constructs positive training sets from sequences where corresponding positions are marked as ‘1’ and negative training sets from sequences where corresponding positions are marked as ‘0’, then separately merges all matched positive and negative sample sequences to form comprehensive training data. This mode is primarily designed to explore broader sequence-taste relationships, facilitating the model's learning of latent space representations for different taste combinations. Subsequently, the LA-VAE undergoes end-to-end training using the filtered training data to learn latent space representations of target taste features.”

The corresponding code implementation demonstrates how the system automatically filters training data based on user-defined taste patterns:

During inference, the only input required is the user-defined five-digit taste feature code. The system then automatically constructs appropriate training datasets, trains the LA-VAE model, and generates candidate peptide sequences through latent space sampling and decoding, requiring no additional sequence inputs or manual intervention.

3. Some hyperparameters lack justification, including latent space dimensionality (2000), K value in KNN (k = 5), and the model weights in SpepToxPred. Please explain the rationale or tuning procedure for these parameters.

We appreciate the reviewer’s insightful questions regarding hyperparameter justifications.

First, we would like to clarify our setting for the K value in KNN (k=5). The k=5 in KNN means that for each generated peptide sequence, we select the 5 nearest positive samples and 5 nearest negative samples in the latent space for distance calculation and ranking comparison. This represents a reasonable empirical choice, where the value ‘5’ is analogous to the five-replicate testing commonly used in experimental research, providing sufficient statistical samples for effective differential analysis while avoiding excessive computational complexity. From a technical perspective, k=5 performs well in the bias-variance tradeoff: selecting too few neighbors (such as k=1 or k=2) may lead to unstable results that are susceptible to noise interference; while selecting too many neighbors, though theoretically feasible, would increase unnecessary computational costs and potentially introduce irrelevant distant samples. In our bilateral distance evaluation strategy, k=5 can effectively balance the evaluation accuracy of “positive sample affinity” and “negative sample repulsion,” ensuring that generated peptide sequences possess good discriminative features in the latent space. The rationality of this parameter choice has been validated through wet-lab experiments (Figure 5).

Regarding the hyperparameter tuning and analysis for the LA-VAE model, including the latent space dimensionality (2000), we have systematically organized and compiled the results in Supplementary Information 02.

We have also added the following description at the end of Section 4.2.2 in the manuscript:

“Additionally, for LA-VAE hyperparameter tuning, each hyperparameter configuration was run three consecutive times, with the minimum average Loss_tol of generated sequences used as the evaluation criterion. Details can be found in Supplementary Data 5.”

This supplementary material provides comprehensive justification and tuning procedures for LA-VAE hyperparameters, ensuring transparency and reproducibility of our model development process.

We have already provided comprehensive justification for the model weights in our manuscript. In section 2.3, we clearly described the systematic optimization process: “We further developed a weighted voting-based ensemble learning framework, where the weight configuration (RF: 0.3, LGBM: 0.1, XGB: 0.2, KNN: 0.2, LR: 0.2) maximized prediction performance (MCC = 0.6540, accuracy = 0.8255, precision = 0.8629).” The rationale for weight allocation was explicitly provided: “The highest weight (0.3) of RF in the ensemble model indicated its superior capability in capturing toxicity-related sequence features.”

The weights were determined through comprehensive evaluation based on Matthews Correlation Coefficient (MCC) during 10-fold cross-validation, with systematic testing of different weight combinations using a step size of 0.1. As stated in the manuscript:

“Detailed weight calculation results (step size: 0.1) are available in Supplementary Data 1,” and visual representation is provided in Figure 4, which shows the systematic framework and the optimal weight combinations for the top 5 ensemble models, including SpepToxPred (Model 1). Therefore, the justification and tuning procedure for SpepToxPred model weights have been thoroughly documented in both the main manuscript and supplementary materials, following standard ensemble learning optimization practices.

4. The criterion that “sequences that simultaneously satisfy both ‘positive sample affinity’ and ‘negative sample repulsion’ are selected as final outputs” is unclear. Please provide a quantitative definition of this criterion.

Thank you for pointing out the lack of clarity in our description of the sequence selection criterion. We acknowledge that the original statement was indeed vague and lacked quantitative definition.

In the source code (02_LA-VAE.py), the bilateral distance evaluation strategy is implemented through the following quantitative steps:

- ‘Positive sample affinity’: quantified by minimizing avg_dist_to_positive (average Euclidean distance to 5 nearest positive training samples)
- ‘Negative sample repulsion’: quantified by maximizing avg_dist_to_negative (average Euclidean distance to 5 nearest negative training samples)
- Final selection: top 25% candidates with smallest differential values (diff_avg_dist_pos_neg = avg_dist_to_positive - avg_dist_to_negative)

We have added a detailed quantitative explanation at the end of section 4.2.2, expanding the existing statement about "constructing distance matrices based on Euclidean metrics" into three comprehensive paragraphs that define both concepts and describe the dual-ranking selection strategy with specific mathematical formulations and implementation details. As shown below:

“In taste avoidance mode, the system projects high-dimensional latent space onto two-dimensional manifolds through principal component analysis to characterize positive and negative sample distributions, constructing distance matrices based on Euclidean metrics to provide quantitative criteria for sequence screening.

Specifically, the bilateral distance evaluation strategy computes k-nearest neighbor distances (k=5) for each generated sequence candidate in the latent space. For every candidate sequence z, the system calculates the average Euclidean distance to the five nearest positive training samples (avg_dist_to_positive) and the five nearest negative training samples (avg_dist_to_negative) when negative samples are available. The 'positive sample affinity' criterion is quantified by minimizing avg_dist_to_positive, ensuring generated sequences maintain close proximity to desired taste characteristics in the latent representation. Conversely, the 'negative sample repulsion' criterion is quantified by maximizing avg_dist_to_negative, ensuring generated sequences maintain sufficient distance from undesired taste properties.

The final sequence selection employs a dual-ranking strategy: sequences are sorted first by avg_dist_to_positive in ascending order (prioritizing positive affinity), then by avg_dist_to_negative in descending order (prioritizing negative repulsion). When both positive and negative samples are used, the system calculates a differential metric (diff_avg_dist_pos_neg = avg_dist_to_positive - avg_dist_to_negative) and retains the top 25% of candidates with the smallest differential values, representing sequences that simultaneously demonstrate strong positive sample affinity and effective negative sample repulsion. This quantitative framework ensures precise control over taste feature generation while maintaining computational efficiency through k-nearest neighbor approximation.”

5. The training dataset contains only 1,131 sequences but encompasses many taste combinations. For certain combinations, the number of available sequences may be very small. Please discuss how the model ensures sufficient positive and negative samples for effective learning.

First, our system employs an intelligent positive/negative sample assignment strategy to maximize training effectiveness despite limited data for certain taste combinations. Our LA-VAE model utilizes a sophisticated five-digit encoding system (‘1’ for desired taste, ‘0’ for avoided taste, ‘x’ for no preference) that automatically constructs training sets from the available data. When users input patterns containing ‘0’, the system strategically includes both positive samples (peptides matching desired taste profiles) and negative samples (peptides that don't match the pattern), creating effective contrastive learning scenarios. For example, when targeting sweet peptides while avoiding salty and umami tastes (pattern ‘x1x00’), the system leverages not only the direct sweet peptides as positive samples but also utilizes salty and umami peptides as negative contrasts. This approach is particularly powerful because our dataset contains over 30% multi-taste peptides (including 250 dual-taste and 94 triple-taste peptides), allowing these complex peptides to serve as training samples for multiple taste patterns simultaneously, thereby effectively multiplying our training resources.

Second, our technical innovations compensate for sample limitations through advanced learning mechanisms specifically designed for small dataset scenarios. The LA-VAE framework incorporates a sophisticated loss supervision mechanism and adaptive extension strategy that ensures optimal learning under limited data conditions. Our dynamic loss monitoring strategy precisely tracks and preserves optimal solutions during training by continuously comparing performance across training epochs and automatically saving the best-performing model states. When standard training fails to achieve expected improvements, the elastic extension mechanism automatically triggers additional training cycles, continuing to explore optimal solutions without manual intervention. Furthermore, the variational autoencoder architecture captures continuous distributions in latent space rather than discrete classifications, enabling the model to learn generalizable taste-structure relationships from limited samples. The experimental validation demonstrates the effectiveness of this approach: we successfully designed and validated 73 novel functional peptides with expected taste properties and excellent safety profiles (cell viability >90%, hemolysis <1.5%), thus proving that our technical framework effectively overcomes the limitations imposed by small sample sizes for certain taste combinations.

We have added a discussion at the end of the second paragraph in section 3. Discussion regarding how our model ensures sufficient positive and negative samples for effective learning:

“Furthermore, our intelligent positive/negative sample assignment strategy, combined with the loss supervision mechanism and adaptive extension approach, provides an effective technical approach to mitigate the challenge of limited samples for certain taste combinations by strategically leveraging multi-taste peptides as training resources across different taste patterns and ensuring optimal learning convergence under small dataset conditions.”

6. The current validation is insufficient, as the study only demonstrates the generation of bitter-avoidance peptides. It is recommended to include simpler baseline experiments, such as generating peptides with a single taste or with simple dual-taste combinations, and report the success rate.

We appreciate the reviewer's valuable suggestion regarding the breadth of experimental validation. We acknowledge that more extensive and diversified experiments would indeed provide stronger evidence than a single experimental set. However, please allow us to provide the following clarifications.

- Regarding the scale and robustness of our current validation:

Our experimental validation involved the synthesis and testing of 73 peptides, which represents a substantial validation scale for taste peptide research. Each peptide underwent comprehensive evaluation including electronic tongue analysis at two concentrations (0.1 mg/mL and 1.0 mg/mL), cytotoxicity assessment across four human cell lines (HPNE, HEK293T, HUVEC, and BEAS-2B), and hemolysis testing using mouse red blood cells. The electronic tongue analysis revealed that all 73 peptides successfully exhibited the target taste properties: sweet and umami characteristics at 0.1 mg/mL, with universal salty response emerging at 1.0 mg/mL, demonstrating consistent and reproducible taste profiles across the entire validation set.

- Regarding experimental constraints and resource considerations:

The synthesis of peptides presents significant logistical and financial challenges. The synthesis of merely 20 peptides requires approximately one month of processing time using solid-phase peptide synthesis (SPPS) methodology. The electronic tongue sensors used in our taste evaluation are expensive consumables, and testing our 73 peptides has exhausted the current laboratory's sensor inventory, necessitating procurement of new sensors at a cost of several thousand dollars. These resource limitations represent common challenges in experimental peptide research that must be balanced against the scope of computational validation.

- Regarding the methodological significance of our contribution:

As a methodological paper focused on computational platform development, our experimental validation provides sufficient evidence for proof-of-concept demonstration. The 73 validated peptides not only confirm TastePepAI's capability but also significantly expand the existing taste peptide library, providing valuable molecular resources for the research community. The consistency of results across all tested peptides strongly supports the reliability of our computational approach.

- Regarding future validation plans:

We recognize the importance of continued validation and are actively planning more comprehensive experimental studies to further enhance TastePepAI's performance and reliability. However, such extensive validation efforts will require substantial time (several years) and resources due to the complexity of taste peptide synthesis, purification, and sensory evaluation procedures.

In conclusion, while we acknowledge that broader experimental validation would be ideal, our current validation with 73 peptides provides robust evidence for the effectiveness of TastePepAI as a computational platform for taste peptide design, particularly given the methodological focus of this work and the substantial resource requirements for peptide synthesis and evaluation.

7. While the experimental verification is commendable, demonstrating the validity of this computational design method based solely on a single experimental set may be insufficient. Ideally, the method should be systematically evaluated using independent test sets and quantitative metrics (e.g., sequence recovery rates analogous to protein design studies). If this is challenging, at minimum, a confidence or uncertainty measure for the generated sequences should be provided to allow users to assess their reliability.

Thank you for this valuable suggestion regarding the systematic evaluation of our computational design method. We appreciate your thoughtful consideration of evaluation metrics that could strengthen the validation of TastePepAI’s effectiveness.

Regarding independent test sets, we respectfully argue that the 73 peptides selected for experimental validation in our study essentially constitute an independent test set for evaluating the generative performance of TastePepAI. These peptides were computationally generated de novo by LA-VAE and subsequently subjected to rigorous experimental validation, including electronic tongue analysis, cytotoxicity assessment, and hemolysis testing. The results demonstrated that all 73 peptides exhibited the desired taste properties (sweet, umami, and salty characteristics) while maintaining excellent safety profiles, with cell viability above 90% and hemolysis rates below 1.5%. This experimental validation provides direct evidence of our method's effectiveness. We acknowledge that additional independent test sets would be valuable for further validation; however, our comprehensive training dataset already encompasses nearly all taste peptide sequences available in public databases and literature that meet our criteria. Creating truly additional independent datasets would require generating and experimentally validating entirely new peptide sequences.

Regarding sequence recovery rates, to our knowledge, this metric refers to the ability of protein design methods to correctly recover (or predict) specific residues (amino acids) in natural protein sequences. This indicator is primarily used to evaluate whether model-designed proteins are closer to natural proteins and has been widely employed by many protein sequence design algorithms such as LigandMPNN (10.1038/s41592-025-02626-1), CarbonDesign (10.1038/s42256-024-00838-2), and ProteinMPNN (10.1126/science.add2187). However, TastePepAI is a short peptide generative model, not a protein design model. We have referenced evaluation methods from similar peptide design models. For instance, Huang et al. (10.1038/s41551-022-00991-2) conducted sequence and physicochemical property analyses comparing their generated sequences with known antimicrobial peptides, demonstrating that their generated peptides exhibited low sequence similarity but high physicochemical property similarity compared to known peptides, indicating good model performance. We adopted a similar approach (Figure S6). More importantly, we monitored the loss dynamics and spatial distribution of positive and negative datasets during the training process (Figure S5). Our comprehensive training monitoring revealed three critical phases: during the early phase (epoch 10), although the total loss remained relatively high (${Loss}_{tol}=7.5719$ for positive training and ${Loss}_{tol}=0.7356$ for negative training), the model demonstrated rapid convergence. In the optimization phase, the model achieved optimal performance (${Loss}_{tol}=0.2088$ for positive training and ${Loss}_{tol}=0.1720$ for negative training), followed by the global optimization phase where even lower loss values were attained (${Loss}_{tol}=0.1817$ for positive training and ${Loss}_{tol}=0.1658$ for negative samples). Both ${Loss}_{rec}$ and ${Loss}_{KL}$ exhibited synchronized reduction throughout the training process, demonstrating the model’s progressive learning of taste-sequence associations. Furthermore, our latent space analysis showed that generated samples initially displayed distinct separation from positive samples, followed by gradual convergence and substantial overlap with the positive sample distribution, confirming the model's effective learning of target taste properties while maintaining the capability for novel sequence generation. Consequently, we believe that the dynamic loss monitoring, spatial distribution tracking, and the evaluation of sequences and physicochemical properties provide a comprehensive assessment of our model's effectiveness in generating taste-active peptides.

Regarding confidence or uncertainty measures, we would like to clarify that TastePepAI already incorporates a confidence assessment mechanism through our dual-side distance evaluation strategy in the latent space. Specifically, our LA-VAE model calculates the average distance from generated peptides to positive taste-active samples (avg_dist_to_positive) and evaluates the spatial distribution overlap between generated sequences and known taste peptides. As described in our manuscript and demonstrated in Figure 3 and S5, we monitor the latent space distribution during training, where generated samples initially show distinct separation from positive samples but gradually converge and achieve substantial overlap with the positive sample distribution. This spatial proximity in the latent space serves as an inherent confidence measure - peptides that cluster closer to known taste-active peptides in the latent space exhibit higher confidence for possessing the desired taste properties. Our k-nearest neighbor-based selection strategy essentially functions as a confidence-driven filtering mechanism, prioritizing peptides with minimal distances to validated taste-active sequences. The 73 experimentally validated peptides were selected based on this spatial proximity criterion, which explains their consistently successful experimental validation with high success rate for taste activity and excellent safety profiles (Figure 5 and S7).

Furthermore, inspired by your valuable suggestion, we have enhanced our confidence assessment by incorporating explicit VAE-based uncertainty measures. We have implemented the following key modifications in our updated source code (02_LA-VAE_uncertainty_quantification.py):

(1) Added VAE uncertainty quantification method:

(2) Enhanced sequence generation with confidence scoring:

(3) Integrated confidence metrics in distance calculations:

(4) Updated output format with confidence information:

These modifications provide users with explicit numerical confidence scores (0-1 range) for each generated peptide, where higher scores indicate lower uncertainty in the VAE's latent space representation. We have committed this updated version to our github repository with the following documentation (https://github.com/leleshidawang/TastepepAI).

8. The term “losstol” appears multiple times but is not defined. Please provide a clear explanation.

Thank you for pointing this out. The issue of undefined "losstol" has been resolved through the comprehensive terminology standardization described in our response to your first comment. The term "losstol" has been unified as “${Loss}_{tol}$” (total loss) throughout the manuscript, with clear mathematical definition and explanation provided in Section 4.2.2.

“The total loss function of LA-VAE comprises two main components with equal weighting:

$${Loss}_{tol}={Loss}_{rec}+{Loss}_{KL}$$

The reconstruction loss employs binary cross-entropy with dimensional scaling to balance the contribution relative to the KL divergence term:

$${Loss}_{rec}=\frac{1}{N}\sum_{i=1}^{N} \left[ -\sum_{j=1}^{14} \sum_{k=1}^{21} \left[ x_{i,j,k}\log\left( \hat{x}_{i,j,k} \right)+\left( 1-x_{i,j,k} \right)\log\left( 1-\hat{x}_{i,j,k} \right) \right] \right]\times294$$

where $N$ is the batch size, $x_{i,j,k}$ represents the one-hot encoded input for the $i$-th sample at position $j$ and amino acid $k$, $\hat{x}_{i,j,k}$ is the corresponding reconstructed output, and the scaling factor $294=14\times21$ ensures balanced optimization between reconstruction and regularization terms.

The KL divergence loss follows the standard VAE formulation:

$${Loss}_{KL}=-\frac{0.5}{N}\sum_{i=1}^{N} \sum_{d=1}^{2000} \left[ 1+\log\sigma_{i,d}^{2}-\left( \mu_{i,d} \right)^{2}-\exp\left( \log\sigma_{i,d}^{2} \right) \right]$$

where $\mu_{i,d}$ and $\log\sigma_{i,d}^{2}$ are the mean and log-variance outputs from the encoder for the $d$-th latent dimension of the $i$-th sample, respectively. Both loss components utilize equal weighting (1:1 ratio) to ensure balanced optimization between reconstruction fidelity and latent space regularization.”

This standardization ensures consistent nomenclature and eliminates the previous ambiguity regarding this terminology.

9. The description of the dataset (sources, size, preprocessing and filtering criteria) should be provided in the Methods section. The Results section can present statistical characteristics or distributional analyses.

Thank you for this important structural suggestion regarding the organization of dataset information. We have reorganized the manuscript to clearly separate methodological details from statistical analyses as recommended.

We have revised the opening paragraph of Results 2.1 to present only distributional analyses and statistical findings:

- Current version:

"The taste peptides demonstrate a clear predominance of short sequences, with 88.54% of the collected peptides not exceeding 10 amino acids in length, while sequences of 15 amino acids or longer constitute less than 3% (Figure 2A). Regarding taste type distribution, the curated dataset exhibits significant imbalance: umami (575) and bitter (541) peptides dominate, while sour (201), sweet (162), and salty (141) peptides are relatively underrepresented (Figure 2B). This distribution pattern likely stems from two key factors: the application potential of umami peptides in food flavor enhancer development has driven related research, while the preferential generation mechanism of bitter peptides during protein proteolysis leads to their prevalence in fermented products. Amino acid composition analysis (Figure 2C) reveals diverse residue distribution patterns among different taste peptides, with sour and umami peptides showing high compositional similarity, while salty and bitter peptides are distinguished by high frequencies of aspartic acid (15.36%) and proline (17.90%), respectively."

Key methodological content removed from Results: The sentence "To reduce data noise and enhance model specificity, we excluded sequences of 15 amino acids or longer (blue columns, Figure 2A) from subsequent modeling" has been moved to Methods.

We also have expanded Methods 4.1 to include all dataset sources, size, preprocessing, and filtering criteria:

- Current version:

“Taste peptide data were comprehensively collected through multiple channels. Initially, data were extracted from specialized taste peptide databases, including BIOPEP-UWM and TastePeptidesDB. Subsequently, we integrated datasets from established taste peptide prediction models (such as Umami-MRNN, VirtuousUmami, and IUP-BERT). Additionally, extensive literature searches were conducted on PubMed and Google Scholar using combinations of ‘Tastes’, ‘Sour’, ‘Sweet’, ‘Bitter’, ‘Salty’, ‘Umami’ with ‘Peptides’ as search keywords. During data preprocessing, sequences containing non-standard amino acid residues were removed, and cases where the same sequence was reported with different taste properties across multiple studies were handled uniformly by annotating them as possessing all reported taste properties. Following initial processing, 1161 peptide sequences were obtained. To reduce data noise and enhance model specificity, sequences of 15 amino acids or longer were further excluded, ultimately resulting in a training dataset comprising 1131 taste peptides.”

Meanwhile, we also deleted the sentence "A total of 6861 toxic and 9183 non-toxic peptides from multiple public databases and literature were integrated (see Methods)." from the first paragraph of section 2.3.

10. Regarding uncertain taste labels (“x”), the paper introduces this notation but does not explain how the model processes such labels.

Thank you for pointing out this important gap regarding the handling of uncertain labels ('x') in our encoding system. We appreciate your attention to this technical detail.

Please allow us to first introduce you to the details of how our model handles 'x' labels. In the 02_LA-VAE.py file from github, for taste peptides, positions marked as '1' require the peptide to possess that specific taste (char == '1'), positions marked as '0' require the peptide to NOT have that taste (char == '0'), and positions marked as 'x' are ignored during the matching process, as shown in the following code:

In fact, after running the code, the program explicitly explains the handling of 'x' to users:

Although the handling of 'x' is quite clear in our code implementation, as you pointed out, we did not explicitly explain how the model processes such labels in the manuscript. Therefore, we have now added explicit explanation of 'x' label processing in section 4.2.1:

"During training data selection, the system processes uncertain labels ('x') through flexible pattern matching: for positions marked as 'x', the model collects peptide sequences containing any taste state ('1', '0', or 'x') at those positions, as 'x' indicates the user has no specific preference or requirement for taste features at that position."

11. Ensure consistent terminology when describing taste categories (e.g., always use the plural form: “sweet, salty, and umami tastes”).

Thank you for pointing out this terminology inconsistency. We will ensure consistent usage throughout the manuscript by using the appropriate singular/plural forms when describing taste categories. Specifically, we will use the plural form "tastes" when referring to multiple taste categories simultaneously (e.g., "sweet, salty, and umami tastes") while maintaining the singular form when describing individual taste types (e.g., "sweet taste" or "bitter taste"). We have carefully reviewed the entire manuscript to standardize this terminology.

Reviewer #3: The paper proposes a computational pipeline for the generation of taste peptides. The main components of the methodology are loss-supervised adaptive variational autoencoder (LA-VAE) for the generation of peptides, combined with a toxicity prediction model. The approach has been validated in vitro (by testing synthesized peptides on cell cultures) and through an electronic tongue. A collection of generated taste peptides has been made available through the development of a public database.

The paper is interesting, methodologically sound and well written. The approach is original and represents a significant advancement of the state of the art. Consequently, my overall opinion on the paper is positive.

I only have a few minor comments:

- The github repository with the source code is available, but the web pages at the domain http://www.wang-subgroup.com/ (which seem to contain a lot of necessary stuff) are not available. When I try to connect (from Europe) I get an error page with text in (probably) Chinese language. Maybe this is a small technical problem, easy to fix (this is why I suggest "minor" revision), but the availability of these web pages is crucial for the acceptance of this paper

Thank you for bringing this important technical issue to our attention. We sincerely apologize for the inconvenience you experienced when accessing our website from Europe.

The Chinese error page you encountered was a notification from Tencent (a major Chinese cloud service provider) indicating that although our website had already been registered with the appropriate government departments, we still needed to complete additional registration procedures specifically with Tencent Cloud (Tencent's cloud server service). We were also puzzled by this situation, as our website had been running normally and accessible for a considerable period prior to this issue.

Your timely feedback was crucial in identifying this problem. We have immediately taken action to complete the required registration procedures with Tencent Cloud to ensure that all website links referenced in our paper are fully accessible to the international research community.

We have now resolved this registration issue and the website should be accessible again. If you don't mind, could you please try accessing our website (http://www.wang-subgroup.com/) once more? If you continue to experience any access problems, please do not hesitate to contact us, and we will investigate further to ensure complete accessibility for all users.

We appreciate your patience and understanding regarding this technical issue, and we are committed to maintaining the accessibility of all computational resources mentioned in our manuscript.

- Why the "all_test.fast" file in the github repository contains only very short sequences?

Thank you for this clarification question. The "all_test.fast" file in the github repository contains short peptide sequences that were previously generated using our 02_LA-VAE.py script. These sequences represent sweet and umami taste peptides and serve as a quick testing dataset for users who want to rapidly evaluate and validate our code functionality.

The short sequences in this file are intentionally designed to:

- Provide a lightweight testing dataset that allows users to quickly run and verify our pipeline
- Demonstrate the expected input/output format for our LA-VAE model

We acknowledge that this may have been unclear to users. To address this, we have added comprehensive documentation in the github repository explaining the purpose and contents of the "all_test.fasta" file, including its role as a quick-start testing dataset for code validation. As shown below:

We appreciate your attention to this detail, as it will help improve the usability and transparency of our computational resource for the research community.

- In the abstract: "to efficiently optimizes" -> "to efficiently optimize" and also "facilitates" -> "facilitate"

- In the introduction: "Here, this paper" sounds weird

Thank you for your careful review and helpful suggestions regarding the language improvements. We have addressed these issues as follows:

- **Regarding the grammatical errors in the abstract:** We have corrected both grammatical issues you identified. Specifically, we have changed "to efficiently optimizes" to "to efficiently optimize" and "facilitates" to "facilitate." These corrections have been implemented in the revised manuscript and are highlighted in blue font for your reference.
- **Regarding the awkward phrasing "Here, this paper":** We agree that this expression sounds unnatural in academic writing. We have revised both instances in the Abstract and the final paragraph of the Introduction section, changing "Here, this paper" to "In this work, we..." This modification provides a more professional and standard academic tone while maintaining clarity about our research contributions.

We sincerely appreciate your meticulous review and constructive feedback, which has significantly improved the clarity and readability of our manuscript.
