# Supplementary material for: TastepepAI: An artificial intelligence platform for taste peptide de novo design
Source: PLoS Comput Biol. 2025 Oct 16;21(10):e1013602. doi: 10.1371/journal.pcbi.1013602 (PMC12543283; doi:10.1371/journal.pcbi.1013602)
Supplement: Attachment — Submitted filename: Response_to_the_editor_and_reviewers_1006_YJD.docx [file pcbi.1013602.s009.docx]

**Journal Requirements:**

1) Please revise your current Competing Interest statement to the standard "The authors have declared that no competing interests exist."

We have revised the Competing Interest statement to the standard format: "The authors have declared that no competing interests exist."

2) Please ensure that the links provided in the Data Availability statement are working.

We have confirmed that all links provided in the Data Availability statement are working properly.

**Reviewers' comments:**

Reviewer's Responses to Questions

Reviewer #1: Yue et al. responded appropriately to the recommendations and revised the manuscript to clarify critical aspects. They also clarified the impact on decision-making or model predictions under other conditions as requested.

We sincerely thank you for your constructive feedback and positive evaluation of our revisions. We appreciate your recognition that we have appropriately addressed the recommendations and clarified the critical aspects of our work, including the impact on decision-making and model predictions under different conditions. Your insights have greatly improved the quality and clarity of our manuscript.

Reviewer #2: The authors have addressed my concerns regarding the inference stage by clarifying the input format and providing details on how training data are selected and processed based on user-defined patterns. However, one aspect remains insufficiently clear. In the revised text, the inference stage is described as: “Subsequently, the LA-VAE undergoes end-to-end training using the filtered training data to learn latent space representations of target taste features.” While this explains how the model learns latent representations, it does not specify how latent vectors are subsequently sampled and decoded into candidate peptide sequences. I suggest that the authors provide additional details on the inference workflow, particularly how sampling is performed in the latent space and how these samples are transformed into peptide sequences.

Thank you for acknowledging our previous responses and for your valuable feedback regarding the inference stage. We appreciate your constructive suggestions for improving the clarity of our methodology.

In response to your specific concern about the inference workflow, particularly regarding sampling in the latent space and the transformation of samples into peptide sequences, we have added a detailed description in Section 4.2.2. The following paragraph has been inserted after the training process description:

"Following successful training, new latent vectors are sampled from the learned 2000-dimensional Gaussian latent space using standard normal distributions, representing potential peptide encodings with target taste properties. These sampled latent vectors are subsequently processed through the trained decoder network, which transforms each latent representation into probability distributions over the 20 standard amino acids at each of the 14 sequence positions. Final peptide sequences are constructed by selecting the amino acid with highest probability at each position, effectively converting continuous latent representations into discrete peptide sequences suitable for further evaluation and experimental validation."

This addition provides the technical details you requested, clarifying how the inference process transforms abstract latent space representations into concrete peptide sequences through systematic sampling and decoding procedures.

Reviewer #3: The authors addressed my comments in a satisfactory way in the new version of the paper.

Thank you for your careful review and for confirming that we have satisfactorily addressed your comments. We appreciate the time and effort you invested in reviewing our manuscript, which has resulted in a much stronger paper.
